# Laparoscopic Anterior 180° Partial Fundoplication in Situs Inversus Totalis: Technical Considerations and Literature Review

**DOI:** 10.7759/cureus.94924

**Published:** 2025-10-19

**Authors:** Sze Li Siow, Febra Siam

**Affiliations:** 1 Department of Surgery, Sarawak General Hospital, Kuching, MYS; 2 Department of Surgery, Taylor's University School of Medicine, Subang Jaya, MYS; 3 Faculty of Medicine and Health Sciences, Universiti Malaysia Sarawak, Kota Samarahan, MYS; 4 Department of Surgery, Timberland Medical Centre, Kuching, MYS

**Keywords:** anterior partial fundoplication, gastroesophageal reflux disease, hiatal hernia, laparoscopic fundoplication, mirror-image anatomy, situs inversus totalis

## Abstract

Situs inversus totalis (SIT) is a rare congenital condition characterized by complete mirror-image transposition of thoracic and abdominal organs. Gastroesophageal reflux disease (GERD) secondary to hiatal hernia in such patients presents unique diagnostic and technical challenges for laparoscopic surgery. Literature review revealed only six previous cases of laparoscopic fundoplication in SIT, all employing Nissen fundoplication techniques. We report a 28-year-old female with SIT and symptomatic GERD who underwent successful laparoscopic hiatal hernia repair with anterior 180° partial fundoplication. The procedure required specific technical adaptations, including mirror-image port placement and modified suturing ergonomics to accommodate the reversed anatomy. Surgery was completed in 98 minutes without complications. At one-year follow-up, the patient achieved complete symptom resolution with excellent functional outcomes. This represents the first reported case of laparoscopic anterior partial fundoplication in SIT, demonstrating the feasibility and safety of this approach in patients with reversed anatomy when appropriate technical modifications are employed.

## Introduction

Situs inversus totalis (SIT) is a rare congenital condition characterized by complete mirror-image transposition of thoracic and abdominal organs [1-3]. While patients with SIT typically have normal organ function and life expectancy, the reversed anatomy creates significant technical challenges during surgical procedures, particularly minimally invasive operations requiring precise spatial orientation and instrument manipulation [1,4].

Gastroesophageal reflux disease (GERD) affects patients with SIT at similar rates to the general population, but diagnosis and surgical management are complicated by the atypical symptom presentation and reversed anatomy [1,4]. Laparoscopic fundoplication has become the standard surgical treatment for medically refractory GERD and symptomatic hiatal hernias, with excellent long-term outcomes reported in normal anatomy patients [5,6].

The increasing prevalence of laparoscopic surgery and global medical tourism predisposes surgeons to encounter patients with rare congenital anomalies such as SIT [2]. Among fundoplication techniques, anterior 180° partial fundoplication offers distinct advantages over complete fundoplication, including reduced postoperative dysphagia, fewer gas-related symptoms, and improved quality of life outcomes while maintaining excellent reflux control [7]. Long-term studies (5-20 years) show Nissen and anterior 180° partial fundoplication achieve comparable satisfaction and durability [8-10]. Nissen offers superior reflux control but more side effects [10], while anterior partial fundoplication provides fewer complications and excellent outcomes [8], with 548 patients over 16 years demonstrating sustained improvement and 5.7% reoperation rates, making it an effective quality-of-life-focused alternative [11].

The existing literature on laparoscopic fundoplication in SIT patients consists primarily of isolated case reports describing Nissen fundoplication [1,4,12-15]. No previous reports have described anterior partial fundoplication for hiatal hernia repair in patients with SIT. We present the first case of laparoscopic hiatal hernia repair using anterior 180° partial fundoplication in a patient with SIT, detailing the technical modifications required, clinical outcomes achieved, and a systematic review of all reported laparoscopic antireflux procedures in SIT patients.

## Case presentation

A 28-year-old Indonesian female with known SIT presented with a one-year history of progressive GERD symptoms, including heartburn, acid regurgitation, chronic cough, hoarseness, epigastric pain, and postprandial bloating. These symptoms significantly impacted her quality of life despite medical management. Her medical history was significant for a previous uncomplicated laparoscopic appendectomy. The physical examination was unremarkable, with a body mass index of 21.5 kg/m^2^, and clinically evident dextrocardia on cardiac auscultation.

Upper endoscopy revealed a sliding hiatal hernia with moderate reflux oesophagitis (Grade B, Los Angeles Classification), Z-line at 34 cm, diaphragmatic impression at 36 cm, and Hill grade IV gastroesophageal valve. Oesophageal manometry demonstrated normal motility patterns with complete bolus clearance. Abdominal ultrasonography ruled out gallstones and confirmed mirror-image visceral anatomy, with right-sided stomach and spleen.

Initial treatment with proton pump inhibitor therapy (vonoprazan 20 mg daily) and lifestyle modifications provided only partial symptom relief over a three-month trial period. Given persistent symptoms, confirmed anatomical abnormalities, and patient preference for definitive treatment, surgical intervention was recommended.

Operative technique

Surgery was performed under general anaesthesia with the patient positioned in modified lithotomy with reverse Trendelenburg positioning. The surgeon stood between the patient’s legs to optimize visualization and instrument handling despite the reversed anatomy.

**Figure 1:**
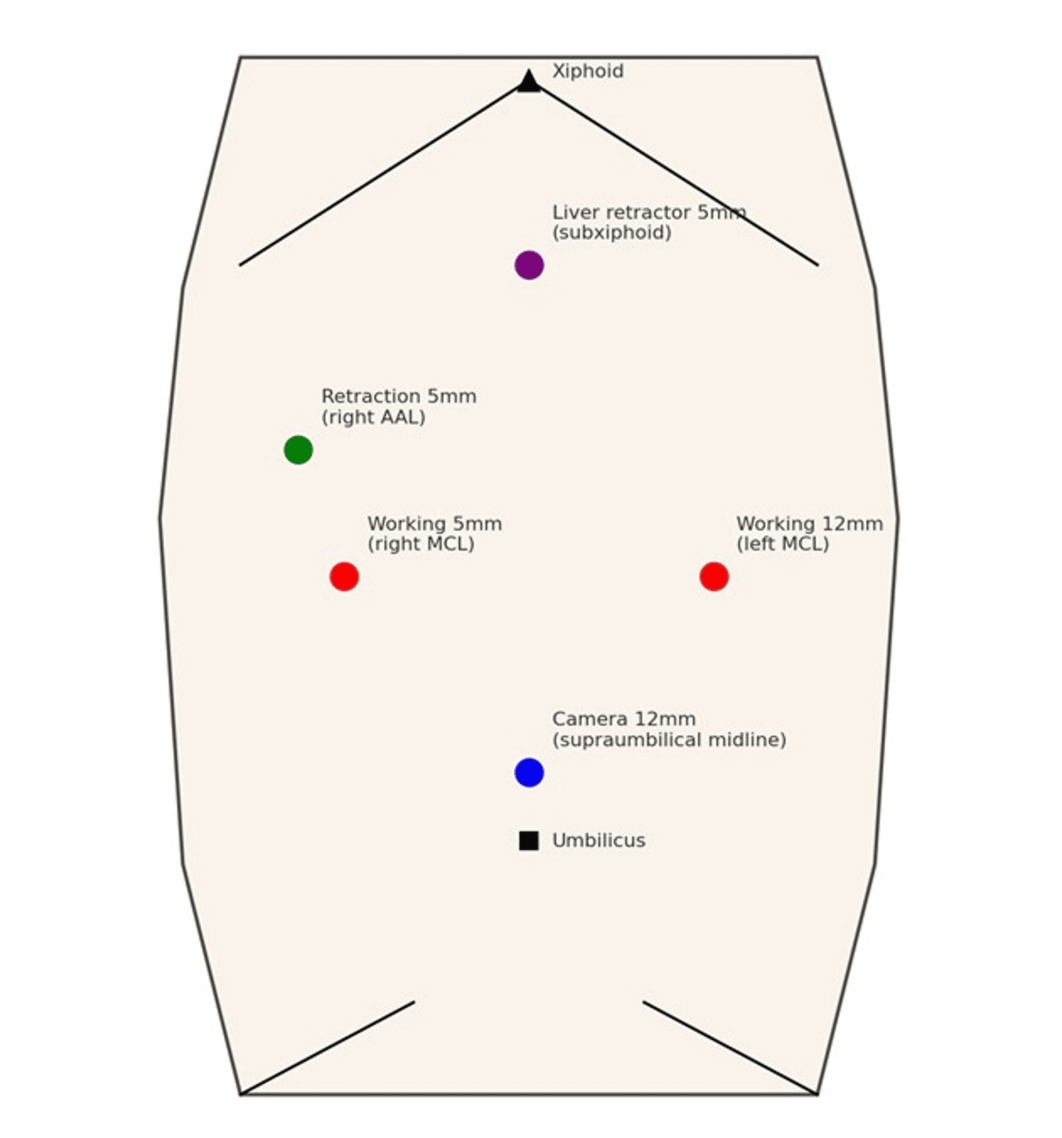
Port placement. AAL: anterior axillary line; MCL: midclavicular line.

A five-port mirror-image configuration was employed to accommodate the reversed visceral anatomy: 12-mm camera port at the supraumbilical midline, 12-mm working port at the left midclavicular line (surgeon’s right hand), 5-mm working port at right midclavicular line (surgeon’s left hand), 5-mm retraction port at the right anterior axillary line, and 5-mm liver retractor port at subxiphoid position (Figure 1).

The operative approach followed standard laparoscopic fundoplication principles with specific modifications for the reversed anatomy. Initial inspection confirmed the mirror-image organ arrangement and absence of adhesions from previous appendectomy. The gastrohepatic ligament was divided using a pars flaccida approach to expose the right crus of the diaphragm. In the context of SIT with left-sided liver positioning, this anatomically right crus was located on the patient's left side. Retroesophageal dissection was performed with careful preservation of the posterior vagus nerves.

Sequential mobilization included division of peritoneal attachments between the diaphragm and fundus, followed by division of attachments between the left crus and oesophagus. The phrenoesophageal membrane was divided to achieve adequate intra-abdominal oesophageal length (≥3 cm), and circumferential mobilization of the gastroesophageal junction was completed.

**Figure 2:**
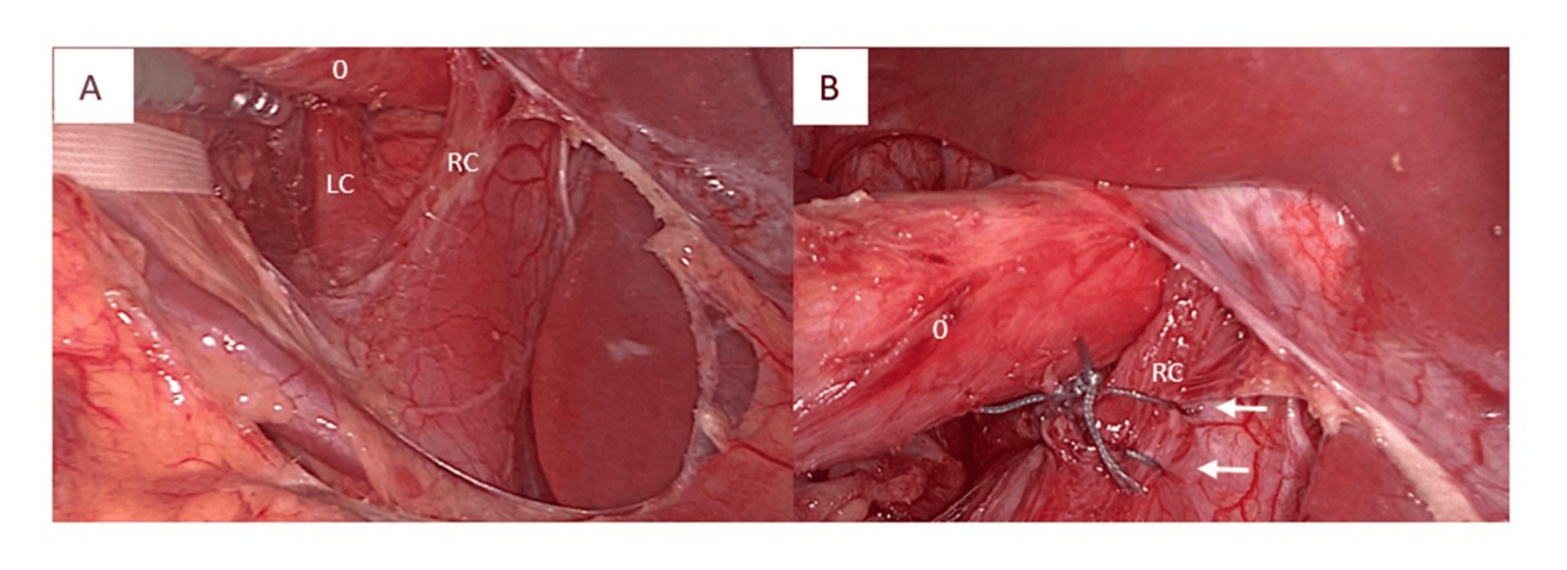
Posterior crural repair. (A) Laparoscopic view showing right crus (RC) and left crus (LC) posterior to the oesophagus (O). (B) Arrows indicate interrupted 2-0 Ethibond sutures for crural closure.

Crural repair was performed using the surgeon's dominant right hand, suturing from the patient's right to left crus with two interrupted 2-0 Ethibond (Ethicon, Cincinnati, OH) (Figure 2), maintaining standard repair principles despite mirrored anatomy. The angle of His was first accentuated using two 2-0 Ethibond sutures: the first securing the fundus to the oesophagus, and the second anchoring the fundus to the left crus and oesophagus.

**Figure 3:**
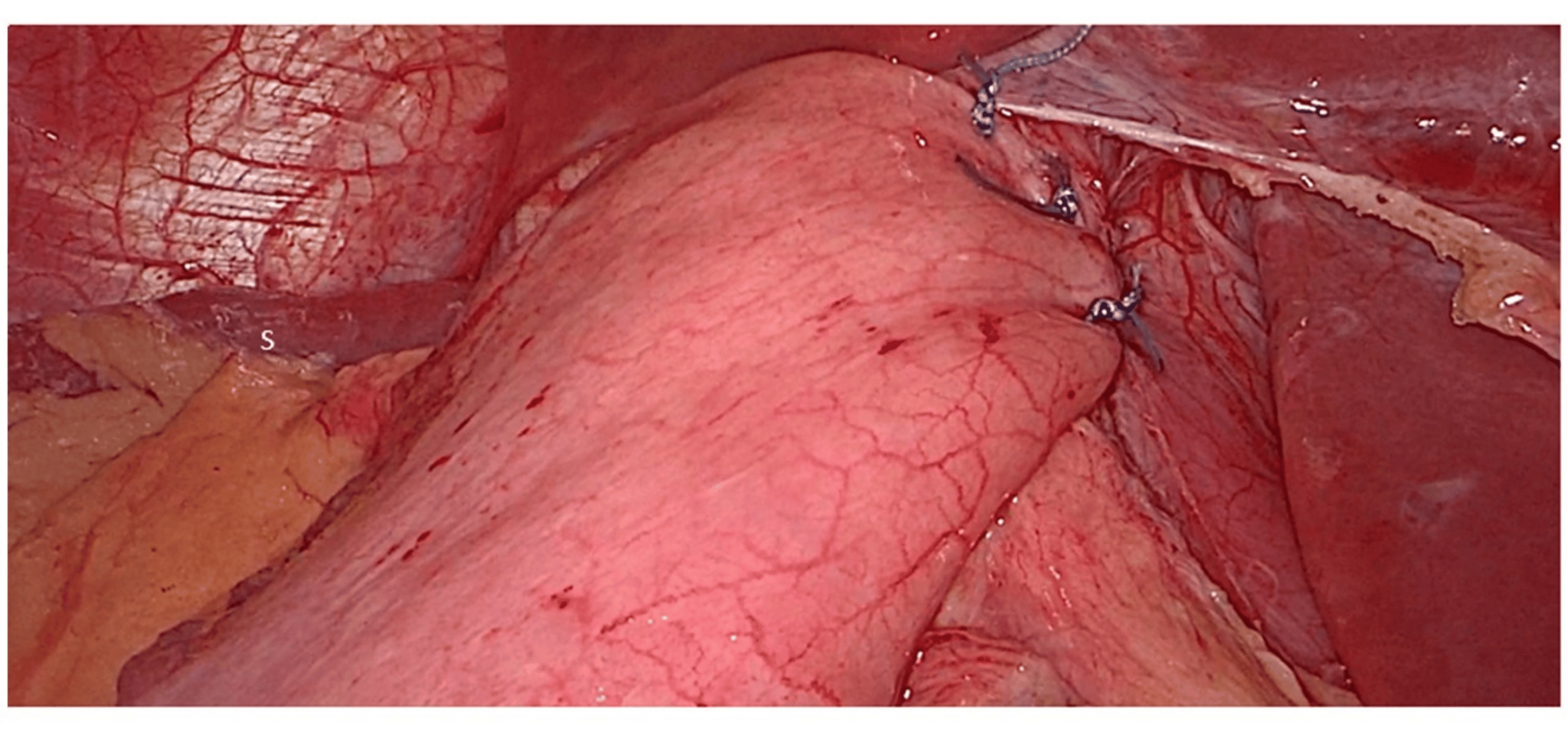
Completed anterior 180° partial fundoplication. Final laparoscopic view showing the completed anterior wrap. The spleen (S) is visible in the background.

The anterior 180° partial fundoplication was constructed using three additional interrupted 2-0 Ethibond sutures (Figure 3). The first suture anchored the fundal apex to the hiatal rim and oesophagus, while the final two sutures completed the anterior wrap by securing the fundus to the left lateral aspect of the oesophagus and right crus.

Operative outcomes

Total operative time was 98 minutes, comparable to standard fundoplication procedures and consistent with reported times for other laparoscopic procedures in SIT patients [4,12-15]. No intraoperative complications occurred, and estimated blood loss was minimal (<50 mL).

Postoperative course and follow-up

The patient recovered uneventfully with standard postoperative care protocols [16,17]. She tolerated clear fluids on postoperative day 0 and progressed to soft diets by postoperative day one. Hospital stay was 24 hours. At one-year follow-up, she reported complete resolution of heartburn, regurgitation, and respiratory symptoms. Mild epigastric bloating was noted but was non-disruptive to daily activities. Patient satisfaction score was 9/10, and she expressed no regret regarding the decision to undergo surgery. She had discontinued all acid-suppression therapy and maintained normal dietary habits without restrictions.

## Discussion

This case represents the first reported laparoscopic anterior 180° partial fundoplication for hiatal hernia repair in a patient with SIT. The successful outcome demonstrates the feasibility and safety of this approach when appropriate technical modifications are employed, expanding the surgical options available for this challenging patient population.

The primary challenges in laparoscopic surgery for SIT patients include altered spatial orientation, modified instrument handling, and adapted surgical ergonomics [4]. Our approach emphasised maintaining surgeon comfort and surgical precision through strategic positioning and port placement. The between-the-legs surgical position proved crucial for maintaining intuitive hand-eye coordination despite the reversed anatomy, a finding consistent with previous reports on fundoplication in SIT [4,12]. The camera assistant stood on the patient's right side, maintaining standard positioning. However, enhanced communication between the surgeon and assistant was essential to ensure proper orientation and optimal visualization of the reversed anatomical landmarks throughout the procedure.

Mirror-image port placement was essential, but we found that maintaining right-handed suturing patterns (surgeon’s right-to-left) was more ergonomically sound than attempting to mirror all surgical movements. This approach allowed for efficient completion of the procedure within normal timeframes.

A systematic review of the literature identified only six previous cases of laparoscopic fundoplication in SIT patients (Table 1) [1,4,12-15]. These cases exclusively employed Nissen fundoplication techniques with operative times ranging from 90 to 110 minutes. Patient demographics included five adults (age 34-65) and two paediatric patients (three months and eight years). All cases reported successful outcomes without major complications, with typical hospital stays of one to two days.

**Table 1:** Published cases of laparoscopic fundoplication in patients with situs inversus totalis. GERD: gastroesophageal reflux disease; HH: hiatal hernia; FTT: failure to thrive; NR: not reported; P: number of ports; BL: between legs position. Good indicates successful symptomatic resolution and no immediate postoperative complications at discharge.

Author, year	Age/sex	Clinical presentation	Procedure type	Technical details	Operative time (min)	Outcome
Hoang et al., 2004 [1]	52/F	Chronic GERD, giant HH	Collis-Nissen	5P, NR	NR	Good
Koo, 2006 [12]	65/M	Chronic GERD	Nissen	5P, BL	110	Good
Tsung et al., 2007 [15]	3 months/F	FTT, persistent emesis	Nissen + gastrostomy	5P, BL	110	Good
Khandelwal et al., 2010 [4]	34/M	Chronic GERD	Nissen	5P, BL	110	Good
Bharatam et al., 2014 [13]	38/M	Sliding HH	Nissen-Rossetti	5P, BL	90	Good
Patel et al., 2014 [14]	8/M	Giant HH, chronic GERD, aspiration pneumonia, FTT	Nissen + gastrostomy	5P, NR	NR	Good
Current case	28/F	Chronic GERD, sliding HH	Anterior 180°partial	5P, BL	98	Good

The consistent success across all reported cases suggests that laparoscopic antireflux surgery is safe and effective in SIT patients when appropriate technical modifications are employed. Our case, with an operative time of 98 minutes and a similar postoperative course, aligns well with these previous reports.

The selection of the anterior 180° partial fundoplication was based on extensive evidence supporting its effectiveness and reduced side-effect profile. Recent meta-analyses demonstrate that partial fundoplication achieves excellent reflux control while significantly reducing the incidence of postoperative dysphagia and gas-related symptoms compared to complete wrap [7,10]. Quality of life studies show sustained improvements across multiple domains with anterior partial fundoplication [16]. The technical simplicity and reduced dissection requirements of partial fundoplication make it particularly well-suited for cases involving complex anatomical variants such as SIT.

This case demonstrates that SIT should not be considered a contraindication to laparoscopic antireflux surgery. With appropriate preoperative planning and intraoperative technical modifications, excellent outcomes can be achieved. The key success factors include a thorough understanding of the reversed anatomy, strategic port placement, maintenance of surgeon ergonomics, and selection of appropriate surgical techniques.

This case report has several limitations. As a single case, it cannot establish definitive safety or efficacy data for anterior partial fundoplication in SIT patients. Long-term follow-up beyond one year will be necessary to assess the durability of the repair and sustained symptom control. Additionally, objective postoperative assessment with pH monitoring or contrast studies would strengthen the evidence for effective reflux control, though the excellent clinical outcomes suggest adequate antireflux function.

## Conclusions

This case represents the first reported laparoscopic anterior 180° partial fundoplication for hiatal hernia repair in a patient with SIT. The procedure was successfully completed with excellent clinical outcomes at one-year follow-up. Key success factors include mirror-image port placement, maintenance of surgeon ergonomics despite reversed anatomy, and appropriate surgical technique selection. With proper technical modifications, laparoscopic antireflux surgery can be safely and effectively performed in patients with SIT. The anterior 180° partial fundoplication technique may offer particular advantages in this population due to its technical simplicity and reduced side-effect profile, though further cases and longer follow-up will be necessary to establish definitive recommendations.
